# Acute suppurative thyroiditis caused by *Streptococcus agalactiae* infection: a case report

**DOI:** 10.1002/ccr3.1048

**Published:** 2017-06-19

**Authors:** Nobuhiro Akuzawa, Toru Yokota, Tsukasa Suzuki, Masahiko Kurabayashi

**Affiliations:** ^1^ Department of General Medicine National Hospital Organization Shibukawa Medical Center 383 Shiroi Shibukawa Gunma 377‐0280 Japan; ^2^ Department of Endocrine Surgery National Hospital Organization Shibukawa Medical Center 383 Shiroi Shibukawa Gunma 377‐0280 Japan; ^3^ Department of Pathology National Hospital Organization Shibukawa Medical Center 383 Shiroi Shibukawa Gunma 377‐0280 Japan; ^4^ Department of Medicine and Biological Science Gunma University Graduate School of Medicine 3‐39‐22 Showa‐machi Maebashi Gunma 371‐8511 Japan

**Keywords:** Acute suppurative thyroiditis, fine needle aspiration biopsy, *Streptococcus agalactiae*, ultrasonography

## Abstract

Acute suppurative thyroiditis is a serious disease; therefore, its diagnosis in the acute phase is important. Fine needle aspiration biopsy of the thyroid gland plays a pivotal role in the diagnosis of acute suppurative thyroiditis. Appropriate culture technique and optimal imaging modalities are also important for its diagnosis.

## Introduction

Acute suppurative thyroiditis (AST), accounting for 0.1%–0.7% of all thyroid diseases, is a rare but potentially fatal bacterial disease 1. AST can result from infection by various microbes, including Gram‐positive aerobes such as *Staphylococcus aureus* and *Streptococcus*, Gram‐negative aerobes, anaerobic microbes, *Pneumocystis jiroveci*, fungi, and *Nocardia* spp. 1. Bacterial infection of the thyroid gland can occur via pyriform sinus fistulae, hematogenous spread, or direct extension of contiguous infection; however, the route or source of infection is not obvious in many cases 1. Common symptoms of AST are anterior neck pain, swelling, and fever; an increased white blood cell (WBC) count, erythrocyte sedimentation rate, or C‐reactive protein (CRP) concentration; and increased thyroid hormone concentrations secondary to destruction of thyroid follicles. However, these findings are not specific to AST, and it is thus important to distinguish AST from malignancy or subacute thyroiditis 1.

We herein present a rare case of AST due to *Streptococcus agalactiae* (*S. agalactiae*) infection. Ultrasonography (US) and computed tomography (CT) of the thyroid gland showed no specific findings of AST, but culture of both blood and thyroid tissue samples obtained by fine needle aspiration biopsy (FNAB) revealed *S. agalactia*e, leading to a definitive diagnosis of AST. To our knowledge, only one case of AST due to *S. agalactia*e infection has been reported in the past 30 years according to the PubMed database 2, suggesting that such cases are extremely rare.

## Case Presentation

An 80‐year‐old Japanese woman was admitted to our hospital because of a 2‐day history of fever. She had no relevant medical history except for hypertension, for which she had been prescribed amlodipine (5 mg/day). She was taking no other medications. She was a nonsmoker and nondrinker. She caught a cold 10 days before admission and recovered 1 week later. Four days before admission, she experienced general malaise without a fever. Two days before admission, she suddenly developed a fever (38.7°C) with left ventral neck pain. She was examined by her home doctor and prescribed oral cefdinir (300 mg/day), but her fever persisted. She was then admitted to our hospital.

**Figure 1:**
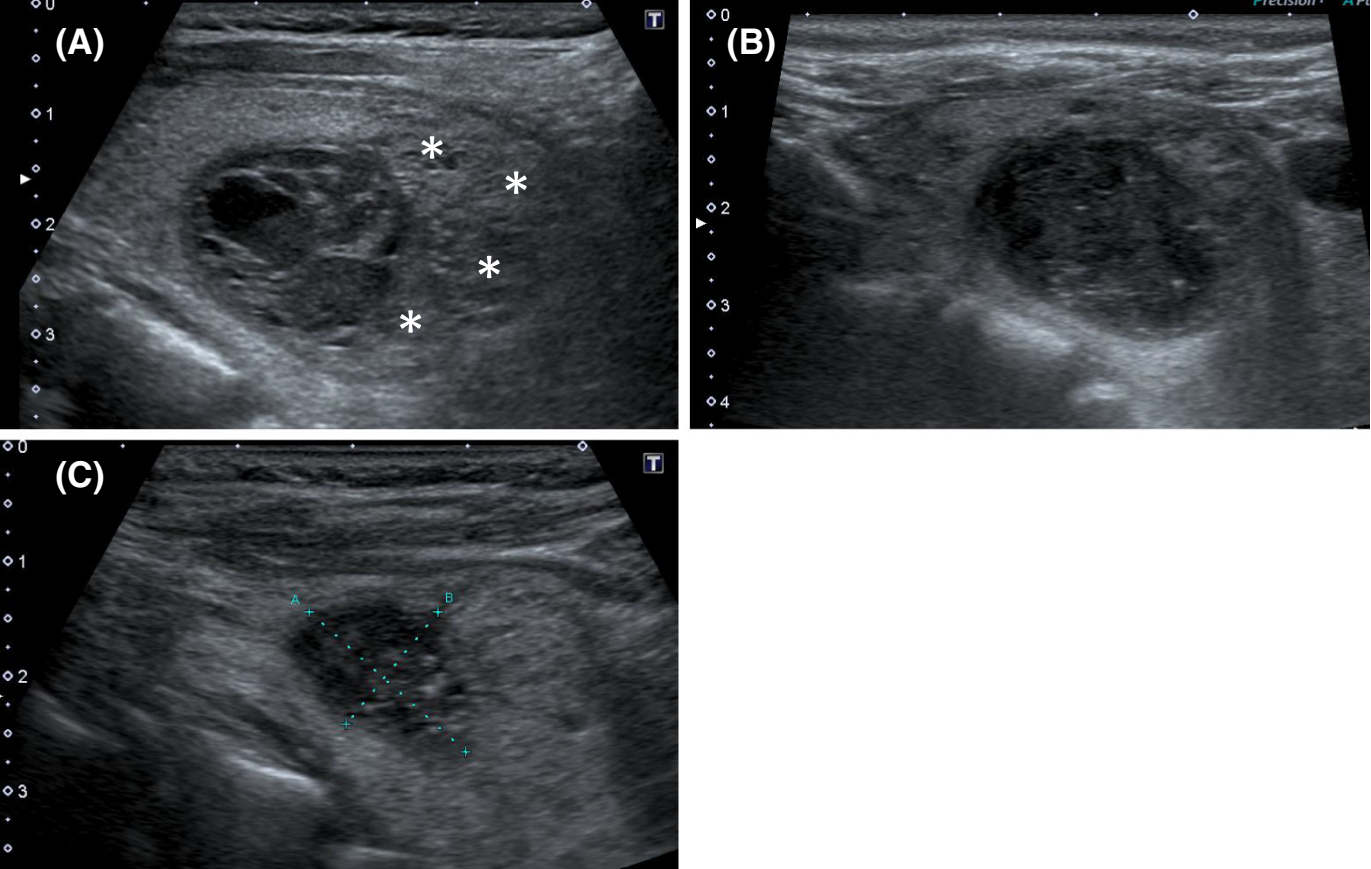
Ultrasonography of the left lobe of the thyroid gland. (A) On admission, multiple preexisting multigoiter nodules were observed (asterisks). Notably, a round, hypoechoic lesion with heterogeneous internal echogenicity was observed. This lesion lacked a distinct capsule and measured approximately 26 × 22 mm in diameter. (B) Ultrasonography on day 6. The hypoechoic lesion had increased in size (29 × 27 mm), and its internal echogenicity was lower than that at the time of admission. (C) Ultrasonography 1 month after discharge. The size of the hypoechoic lesion had become obviously diminished (17 × 15 mm).

On admission, her height was 141 cm, weight was 47 kg, body temperature was 38.0°C, and blood pressure was 126/62 mmHg. Her heart rate was 90 beats/min with a regular rhythm. A physical examination showed no major abnormalities except for mild and tender swelling of the left anterior region of the neck. Chest and abdominal X‐ray films and an electrocardiogram were normal. Laboratory tests showed a high WBC count of 23.4 × 10^9^ cells/L (reference range, 3.5–9.0 × 10^9^ cells/L), lactate dehydrogenase concentration of 243 U/L (reference range, 124–222 U/L), blood urea nitrogen concentration of 21.1 mg/dL (reference range, 8–20 mg/dL), creatinine concentration of 1.16 mg/dL (reference range, 0.46–0.79 mg/dL), and CRP concentration of 19.41 mg/dL (reference range, 0.00–0.14 mg/dL). The fasting plasma glucose and hemoglobin A1c concentrations were 5.3 mmol/L (reference range, 3.9–5.5 mmol/L) and 5.5% (reference range, <6.0%), respectively, indicating the absence of diabetes. Urinary findings were normal. Rapid antigen tests for influenza, pneumococcus, and mycoplasma were negative. Thyroid function tests showed a decreased thyroid‐stimulating hormone concentration of 0.18 mU/L (reference range, 0.35–4.94 mU/L) and increased free T4 concentration of 3.06 ng/dL (reference range, 0.70–1.48 ng/dL). Ultrasonography revealed an enlarged thyroid gland with a heterogeneous texture and multiple confluent nodules that were not well‐circumscribed and were predominantly hypoechoic, suggesting preexisting adenomatous goiter. Notably, a round, heterogeneous, hypoechoic lesion without a distinct capsule, which was obviously different from the other goiter nodules, was observed in the left lobe of the thyroid gland; it measured approximately 26 × 22 mm (Fig. 1A). Plain CT of the neck also showed multiple nodules in the thyroid gland but provided no further information regarding the inside of the nodules. Based on these findings, US‐guided FNAB targeting a hypoechoic lesion was performed to discriminate between AST and subacute thyroiditis soon after admission. Although FNAB did not yield pus, cultures of the thyroid aspirate needle washout, blood, urine, and sputum were carried out. Although the definitive diagnosis was unclear on admission, oral prednisolone (30 mg/day) was prescribed due to suspicion for subacute thyroiditis. Intravenous administration of aminobenzylpenicillin (8 g/day) was simultaneously performed because the possibility of AST was not completely negated.

**Figure 2:**
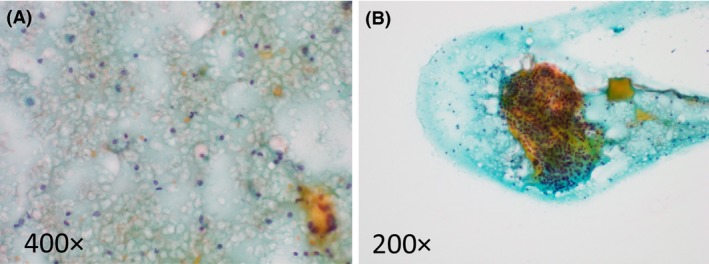
Fine needle aspiration biopsy sample findings. (A) Inflammatory cells including polymorphonuclear leukocytes and lymphocytes were abundant within the exudates around the yielded thyroid tissue. (B) The obtained thyroid tissue showed no remarkable cellular atypia of the follicular epithelial cells.

On day 2, Gram‐positive cocci were detected from the blood culture samples, and administration of the oral prednisolone was discontinued. The patient's general status improved, and she became afebrile on day 4. On day 5, the Gram‐positive cocci detected from the blood culture samples were identified as *S. agalactiae*. Notably, *S. agalactiae* was also detected from the culture samples of the thyroid aspirate needle washout on admission. The urine culture revealed a small amount of *Escherichia coli* (1.0 × 10^4^/mL) and *S. agalactiae* (1.0 × 10^3^/mL); the sputum culture detected no remarkable pathogenic bacteria. All of the *S. agalactiae* strains detected from blood, biopsy needle washout, and urine showed the same antibiotic sensitivity. Gynecologic and urologic evaluations revealed no significant abnormalities. Vaginal secretion culture was performed on day 5, but *S. agalactiae* was not detected. Cytological examination of the FNAB samples on admission revealed an abundance of inflammatory cells, including polymorphonuclear leukocytes and lymphocytes (Fig. 2A), and no remarkable cellular atypia of the follicular epithelial cells (Fig. 2B). Accordingly, we diagnosed the patient with AST due to *S. agalactiae* infection. Ultrasonography of the thyroid gland on day 6 revealed that the hypoechoic lesion inside the left lobe had slightly increased in size (29 × 27 mm) and that the internal echogenicity was lower than that on day 1 (Fig. 1B). The patient's left neck pain resolved and her WBC count normalized on day 10, and her CRP concentration normalized on day 14. Administration of intravenous aminobenzylpenicillin was discontinued on day 14, and she was discharged from our hospital on day 16.

US examination was repeated 1 month after discharge. The size of the hypoechoic lesion was markedly reduced (17 × 15 mm) (Fig. 1C), and the thyroid function test results had also normalized. An otolaryngology evaluation and barium swallow failed to reveal a pyriform sinus fistula. At the time of this writing, the patient had been followed up for 6 months with normal thyroid function and no recurrence of AST.

## Discussion

The thyroid gland is a remarkably infection‐resistant organ because of its high vascularity, lymphatic drainage, tissue uptake of iodine or hydrogen peroxide, and encapsulated structure 3. Factors that may predispose to AST include the presence of a pyriform sinus fistula, third and fourth branchial arch abnormalities, and an immunocompromised status 3. AST may occur in a cystic or degenerated nodule within the thyroid gland 3, but the presence of a causal relationship between adenomatous goiter and AST is unclear. To the best of our knowledge, only three cases of AST in patients with preexisting multinodular goiter, which was also observed in our patient, have been reported in the past 30 years according to the PubMed database 4, 5, 6. These patients were immunocompetent and did not appear to have a pyriform fistula. Notably, two of these three patients developed AST due to hematogenous spread of pathogenic microbes; one developed AST due to an *E. coli* urinary tract infection 4, and the other developed staphylococcal AST due to septic emboli derived from infective endocarditis 5. Meanwhile, another patient developed AST following FNAB 6. In a previous study, the enzymatic antioxidant defense mechanism was impaired in multinodular goiter tissue 7, suggesting that little resistance to oxidative stress may lead to vulnerability of goiter tissue under oxidant‐rich conditions in patients with infection. Accordingly, preexisting multinodular goiter may induce higher susceptibility to bacterial infection of the thyroid gland.

In the present case, the beta‐hemolytic Gram‐positive *S. agalactiae* (Group B streptococcus, GBS) was detected from both the blood and thyroid aspirate needle washout culture samples. GBS is an indigenous bacterium in the urogenital and/or lower gastrointestinal tract and a leading cause of sepsis and meningitis in neonates 8. GBS is also an important cause of morbidity and mortality in elderly and immunocompromised adults; nevertheless, urogenital diseases resulting from Gram‐positive bacteria may be overlooked due to limited culture‐based assays in hospital microbiology laboratories 9. In the present case, the urine culture revealed *S. agalactiae*, indicating that invasive infection of *S. agalactiae* from the urinary tract may lead to bacteremia and subsequent AST. The incidence of GBS bacteremia in people aged >60 years is twice as high as that in patients aged ≤60 years, and the most common comorbidity in patients with GBS bacteremia is reportedly diabetes (25%) 10. Notably, GBS bacteremia can occur in older people as well as in neonates.

Early diagnosis of AST is quite important to avoid a fatal outcome. Typically, US examination in the acute phase shows a hypoechoic lesion spreading in or around the affected thyroid lobe. However, US may show an unclear hypoechoic area in the early acute phase and CT may demonstrate similar low‐density features; this can lead to an erroneous diagnosis of subacute thyroiditis 11. Misdiagnosis of subacute thyroiditis is quite dangerous because prednisolone administration may lead to rapid deterioration of AST 12. Masuoka et al. 11 suggested that the characteristic US findings of AST are a perithyroidal hypoechoic space, effacement of the plane between the thyroid and perithyroidal tissues, and the presence of unifocal hypoechoic lesions. According to their study, the former two are not seen and the latter is less common in patients with subacute thyroiditis. Certainly, the AST lesion was unifocal in the present case, but the former two patterns were not evident. Conclusive evidence of AST in our case was obtained by culture of the aspiration needle washout on admission. Although FNAB is a common procedure for pathological differentiation between AST and subacute thyroiditis 1, our experience also suggests the importance of culture of the thyroid aspirate needle washout as well as histopathological examination of FNAB samples. The limitations of our report include our inability to determine the genetic identity of the *S. agalactiae* strains detected from the blood, biopsy needle washout, and urine and the inability to perform gram staining of the FNAB samples. However, our findings indicate the usefulness of culturing the biopsy needle washout even in patients with AST who show no obvious fluid collection in the thyroid gland; hence, this may also be helpful for early diagnosis of AST.

In conclusion, we have presented a rare case of *S. agalactiae* bacteremia following AST. US‐guided FNAB of the thyroid gland did not yield pus, but a culture of the aspiration biopsy needle washout revealed *S. agalactiae*. Culture could be useful for obtaining a definitive diagnosis of early acute‐phase AST, even in patients with no fluid collection in or around the thyroid gland, because imaging modalities such as US or CT of the thyroid gland may lack the ability to distinguish AST from subacute thyroiditis. Physicians should know the importance of FNAB and culture for the diagnosis of AST in the acute phase.

## Consent

Written informed consent was obtained from the patient.

## Conflict of interest

The authors declare that there is no conflict of interests regarding the publication of this paper.

## Authorship

NA: drafted the manuscript, collected the patient's data, and monitored the patient throughout the whole follow‐up period. TY: collected the patient's data. TS: collected the patient's data. MK: helped to draft the manuscript. All authors have read and approved the final manuscript.
